# Pico-washing: simultaneous liquid addition and removal for continuous-flow washing of microdroplets

**DOI:** 10.1038/s41378-022-00381-3

**Published:** 2022-04-29

**Authors:** Michael J. Siedlik, David Issadore

**Affiliations:** 1 Department of Bioengineering, University of Pennsylvania, Philadelphia, PA 19104 United States; 2 Department of Electrical and Systems Engineering, University of Pennsylvania, Philadelphia, PA 19104 United States

**Keywords:** Engineering, Microfluidics

## Abstract

Droplet microfluidics is based on a toolbox of several established unit operations, including droplet generation, incubation, mixing, pico-injection, and sorting. In the last two decades, the development of droplet microfluidic systems, which incorporate these multiple unit operations into a workflow, has demonstrated unique capabilities in fields ranging from single-cell transcriptomic analyses to materials optimization. One unit operation that is sorely underdeveloped in droplet microfluidics is washing, exchange of the fluid in a droplet with a different fluid. Here, we demonstrate what we name the “pico-washer,” a unit operation capable of simultaneously adding fluid to and removing fluid from droplets in flow while requiring only a small footprint on a microfluidic chip. We describe the fabrication strategy, device architecture, and process parameters required for stable operation of this technology, which is capable of operating with kHz droplet throughput. Furthermore, we provide an image processing workflow to characterize the washing process with microsecond and micrometer resolution. Finally, we demonstrate the potential for integrated droplet workflows by arranging two of these unit operations in series with a droplet generator, describe a design rule for stable operation of the pico-washer when integrated into a system, and validate this design rule experimentally. We anticipate that this technology will contribute to continued development of the droplet microfluidics toolbox and the realization of novel droplet-based, multistep biological and chemical assays.

## Introduction

Droplet microfluidic technology has rapidly developed in the last two decades and is now making a major impact across multiple domains of science and technology, including (1) transcriptomic^1,2^, proteomic^3^, and genomic^4,5^ analyses at the single-cell^6^ and subcellular^7^ levels, (2) ultrahigh-sensitivity clinical diagnostics^8,9^, (3) high-throughput screening for cellular phenotyping^10–13^, directed evolution^14–16^, and materials optimization^17–19^, and (4) high-throughput production of monodisperse microparticles for pharmaceutical, cosmetic, and energy applications^20,21^. Underlying these successes are several fundamental differences between assays carried out in droplets versus conventional, milliliter-scale laboratory apparatuses. By partitioning a fluidic sample into femtoliter-to-picoliter droplets, droplet microfluidics reduces biological and chemical reactions to the micrometer scale and enables the creation of many distinct reaction vessels that can be controlled or analyzed in parallel. Owing to the small sizes of these droplets, mass transport is enhanced with the reduced timescale of diffusion and because of intradroplet circulating flows that occur as a droplet moves through a microfluidic channel, which is an important feature for rapid biomolecular binding assays^22^ and production of monodisperse nanoparticles^23^. When a bulk solution is partitioned into sufficiently more droplets than there are copies of a target analyte, each droplet will likely contain either zero or one target. In this “digital” regime, droplet-based assays can perform absolute quantification with 1000x greater sensitivity and enhanced linearity compared to bulk assays^24,25^ and resolve single cells^26^, organelles^27^, extracellular vesicles^28,29^, and virus particles^30^, as well as individual molecules of nucleic acids^31,32^ and proteins^25,33^. Finally, by arranging droplet unit operations in series on a single chip, a chemical or biological protocol can be carried out automatically and with reduced losses due to manual fluid transfer, thereby reducing reagent costs and experimental time^34^. Development of unit operations, such as droplet encapsulation, merging, splitting, and sorting^35^, has fueled the development of integrated droplet workflows where multiple unit operations can be incorporated in series to produce the desired capability in an automated fashion or in parallel to enhance the number of droplets processed per unit time. Novel droplet unit operations amenable to operating in series and in parallel have the potential to unlock new assays while building upon the successes and harnessing the strengths of droplet microfluidics.

One unit operation that is sorely underdeveloped in droplet microfluidics is washing, i.e., the exchange of fluid through droplets during continuous flow, such as to remove chemical species or perform in-droplet sample preparation^36–38^. Although other bench-scale biological and chemical processes have been successfully mimicked at the microscale, e.g., droplet generation miniaturizes partitioning a fluidic sample into tubes or wells and pico-injection miniaturizes the addition of fluid into existing fluidic compartments^39^, it has proven challenging to create a unit operation that (1) miniaturizes the direct exchange of unwanted fluid with a new fluid and (2) is capable of operating with throughputs demonstrated by other droplet operations, such as generation^20^ and detection^8^. One general approach for realizing this fluid exchange is to alternate (*i*) adding buffer to a droplet via pico-injection or merging that droplet with another droplet and (*ii*) actively or passively splitting the droplet and reducing the concentration of the molecular profile initially contained within the droplet each time the droplet is split^38,40–44^. Alternatively, other techniques transfer the droplet contents from the original droplet into a new droplet^45,46^. While these techniques have pushed the standards for washing efficiency and throughput, and the best performing devices have the ability to perform 10^2^-fold dilutions while operating at 10^2^ Hz^45^, these technologies can suffer from large footprints or the need to synchronize multiple droplets and streams. As a result, it is nontrivial and potentially unfeasible to operate microfluidic chips with these operations arranged in series or in parallel, thereby imposing a challenging limitation for improving washing performance and achieving the >kHz benchmark typical in other droplet operations^8,20,35^.

To address these challenges, we have developed a new approach to droplet washing that is capable of simultaneously adding and removing fluid from droplets with kHz throughput and that can be incorporated robustly with other unit operations, such as droplet generators and other droplet washers. We term this unit operation a “pico-washer” and demonstrate the device architecture required for stable performance. Several key features were necessary to overcome previously unsolved challenges and develop the pico-washer: (1) a fabrication strategy combining interfacial pinning and spatial patterning of channel hydrophilicity to stabilize coflow of adjacent, immiscible phases, (2) an electric field to trigger destabilization of the water–oil–water interfaces and form a fluidic connection between the passing droplets and the pico-washer, and (3) incorporation of in-line flow resistors to allow uniform operation of multiple pico-washers arranged in series. Additionally, we define the process and geometrical parameters that dictate washing performance, create an image processing workflow to characterize the washing process with microsecond-scale and micrometer-scale resolution (provided in the Supplementary Information), and highlight the engineering design rule required for operating multiple pico-washers in series on a single chip. By characterizing this much-needed unit operation and adding it to the droplet microfluidic toolbox, we anticipate that this device will provide a foundation for future development of integrated, multistep droplet workflows.

## Results

### Pico-washer architecture

**Fig. 1:**
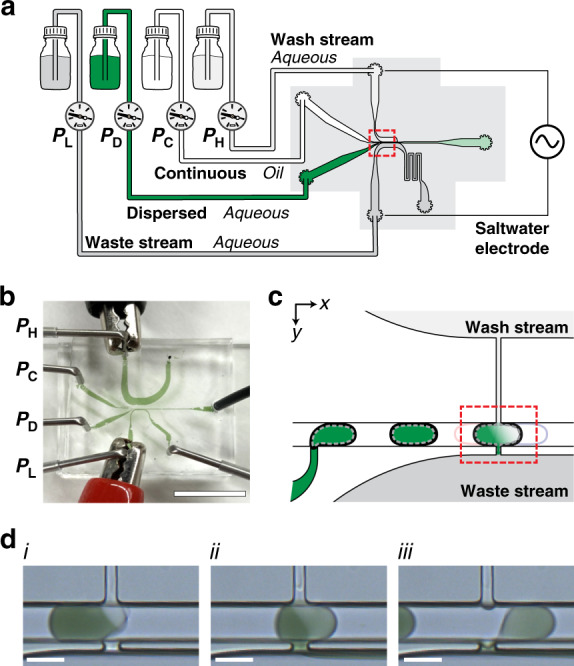
Overview of the pico-washer concept and device. **a** Schematic of the droplet washing strategy described here. **b** Photograph of the microfluidic device with fluidic and electrical connections in place. Pressure notations next to each fluidic input correspond to the streams depicted in (**a**). All fluidic channels and tubing are filled with dye for ease of visualization. Note that the outlet from the wash stream is clamped during operation to direct all fluid through the pico-washer. The scale bar represents 1 cm. **c** Schematic of the droplet generator and pico-washer (highlighted in red) arranged in series in a single microfluidic device. **d** Micrographs of an initially dye-laden droplet (*i*) contacting, (*ii*) fully connected to, and (*iii*) disassociating from a pico-washer. Scale bars represent 50 μm

To develop and evaluate the droplet washing technology, we designed a four-input, pressure-driven microfluidic system that integrated a T-junction droplet generator with a region that we term a “pico-washer” (Fig. 1a–c). Two of these fluidic inputs provided the aqueous dispersed phase and oil continuous phase to the T-junction, which was designed to produce 75 μm diameter water-in-oil (W/O) droplets with a volume fraction of ~0.5. Downstream from the T-junction droplet generator, droplets flowed in plug-flow in a channel that was 50 μm wide and 60 ± 5 μm high. Further downstream was the pico-washer, which consisted of two lithographically defined channels arranged perpendicularly to, and on either side of, the emulsion channel. One channel, 500 μm long by 15 μm wide by 50 μm high, connected the emulsion channel with a high-pressure (“wash”) aqueous stream. The other channel, 15 μm long by 15 μm wide by 50 μm high, connected the emulsion channel with a low-pressure (“waste”) aqueous stream.

**Fig. 2:**
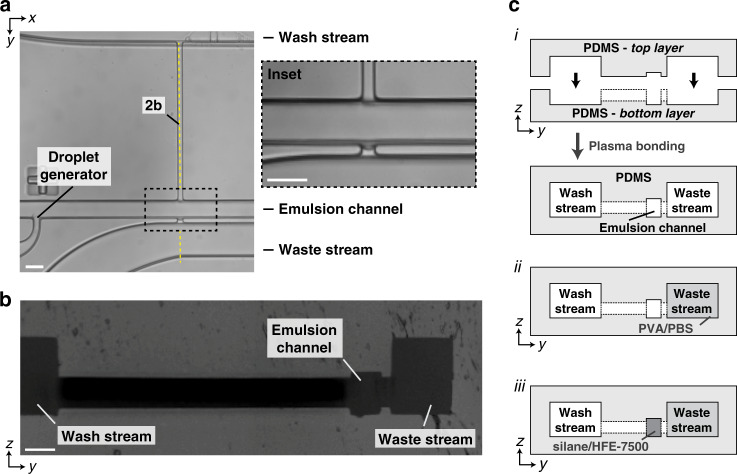
Fabrication of the pico-washer microfluidic device. **a** Micrograph depicting a top-down view of the PDMS microfluidic device. The inset shows a magnified view of the fluid transfer region. **b** Micrograph depicting the cross-section of a PDMS device along the dashed yellow line marked in (**a**). **c** Schematic of the fabrication strategy for making the microfluidic devices. (*i*) First, an entirely PDMS device is constructed by aligning and plasma bonding two lithographically defined pieces of PDMS. Then, (*ii*) the waste channel is rendered hydrophilic, and (*iii*) the emulsion channel is rendered hydrophobic. All scale bars represent 50 μm

We designed the pico-washer so that as a moving W/O droplet made fluidic contact with the two channels of the pico-washer, one at a higher pressure and one at a lower pressure relative to the droplet, fluid would be transferred through the droplet. This was first evaluated qualitatively in washing droplets initially laden with a food coloring dye (Fig. 1d). Each droplet generated by the upstream T-junction transited the pico-washer and underwent the following operations: (1) the moving droplet made fluidic contact with the wash and waste streams (Fig. 1d, *i*); (2) the concentration of dye in the droplet was visibly reduced with continued addition of clear phosphate-buffered saline (PBS) solution and removal of dye solution (Fig. 1d, *ii*); (3) the droplet disconnected from the pico-washer after ~0.5 ms of washing and continued flowing through the emulsion channel (Fig. 1d, *iii*). In these experiments, fluidic connection between the pico-washer and droplets was enabled by an AC voltage (100 V_pp_ at 100 kHz) delivered via a saltwater electrode through the aqueous wash and waste streams and applied across the width of the 50 μm emulsion channel.

### Pico-washer design: interfacial pinning and spatial patterning of channel hydrophilicity

To realize the pico-washer operation experimentally, microfluidic devices were developed and constructed entirely out of polydimethylsiloxane (PDMS) in a multistep process that combined soft lithography and spatial patterning of channel hydrophilicity (Fig. 2). First, microfluidic channels for all fluidic streams were lithographically defined in PDMS (Fig. 2a). Next, a fabrication strategy was developed in which two pieces of PDMS, one for the top of the microfluidic device and the other for the bottom of the device, were aligned and irreversibly plasma bonded to create a device architecture in which the wash, emulsion, and waste streams were taller than the perpendicular channels in the pico-washer that connected these streams (Fig. 2b). The function of this height difference was to introduce a “step” that would promote pinning of the oil–water interface between the continuous phase and waste stream in the *z*-direction, similar to that used to stabilize interfaces in the *xy* plane in other microfluidic systems^47^. Following PDMS device assembly, the waste and emulsion channels were rendered hydrophilic and hydrophobic, respectively, to further promote oil-water interfacial stability (Fig. 2c).

**Fig. 3:**
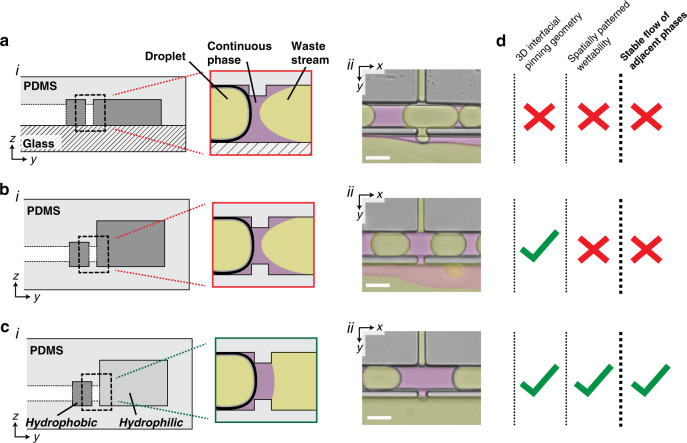
3D interfacial pinning and spatial patterning of surface hydrophobicity are required for stable device operation. **a** (*i)* Cross-sectional schematic and (**a**) (*ii*) micrograph of an in-use microfluidic device that was created by bonding PDMS to a glass microscope slide. **b** (*i*) Cross-sectional schematic and (**b**) (*ii*) micrograph of an in-use, entirely PDMS microfluidic device engineered to have 3D interfacial pinning and rendered entirely hydrophobic. **c**, (*i*) Cross-sectional schematic and (**c**) (*ii*) micrograph of an in-use, entirely PDMS microfluidic device engineered to have 3D interfacial pinning and spatially patterned surface hydrophobicity. False color is added to all micrographs for visualization of aqueous (yellow) and oil (magenta) phases, and each micrograph depicts device operation without any applied electric field. The coordinate system corresponds to that used in Fig. 2: *x* and *y* represent the in-plane dimensions parallel and perpendicular, respectively, to the length of the channel. **d** Summary table describing the features and outcome of each fabrication strategy. All scale bars represent 50 μm

Both interfacial pinning and spatial patterning of surface hydrophilicity were required for stable flow of the W/O emulsion adjacent to the aqueous wash and waste streams contained within the pico-washer. A common failure mode for microfluidic devices that include multiphasic flows of immiscible liquids is destabilization of the interface between the phases. If the pico-washer did not include interfacial pinning or patterned hydrophilicity, this destabilization would lead to a loss of oil from the emulsion channel to the waste channel. Were this loss of the continuous phase to occur, the distance between droplets would decrease, leading to two undesirable outcomes: unstable device operation and merging of successive droplets. This was observed in devices created by “standard” microfluidics soft lithography, in which one lithographically defined piece of PDMS was bonded to a glass slide and the entire device was made hydrophobic (Fig. 3a, *i*), as well as in devices with channel geometries that promoted interfacial pinning but did not feature patterned hydrophilicity (Fig. 3b, *i*). In both cases, oil wetted the adjacent waste channel, resulting in loss of the continuous phase from the emulsion channel (Fig. 3b, *ii* and c, *ii*). However, when the waste and emulsion channels were made hydrophilic and hydrophobic (Fig. 3c, *i*), respectively, the oil phase was confined to the emulsion channel during device operation (Fig. 3c, *ii*). The result of these design iterations was a microfluidic system in which a stream of W/O droplets could stably flow adjacent to the aqueous channels contained within the pico-washer (Fig. 3d).

### Visualization and quantification of pico-washer performance

**Fig. 4:**
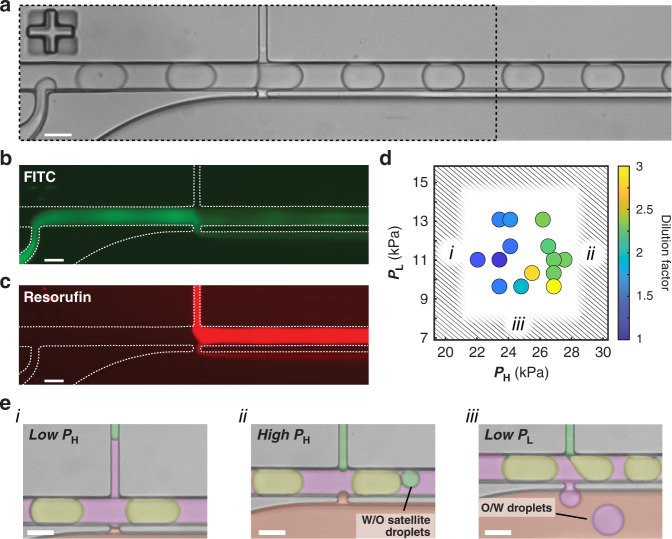
Pico-washer performance depends on the wash and waste stream fluidic pressures. **a** Transmitted light micrograph of a pico-washer device in operation. **b** Fluorescence micrograph of dye initially present within the droplets. **c** Fluorescence micrograph of dye added to droplets during the washing process. **a**–**c** all correspond to the same device. The black dashed box in **a** represents the region depicted in (**b**) and (**c**). **d** Plot of washing efficiency as a function of input pressures to the wash (*P*_H_) and waste (*P*_L_) streams when *P*_C_ = 28 kPa and *P*_D_ = 24 kPa. The color of each data circle indicates the washing efficiency when the device was operated with those input pressures. Regions highlighted by (*i*), (*ii*), or (*iii*) correspond to those depicted in (**e**). **e** Micrographs depicting failure modes that arise when the fluidic pressures are imbalanced: (*i*) the wash stream pressure is too low; (*ii*) the wash stream pressure is too high; and (*iii*) the waste stream pressure is too low. False coloring is added for visualization of wash (green), dispersed (yellow), oil (magenta), and waste (red) streams. All scale bars represent 50 μm.

To visualize and quantify fluid transfer through the droplets, separate fluorescent dyes were incorporated into the dispersed phase and wash stream and simultaneously imaged during device operation (Fig. 4a–c). By capturing long-exposure (>400 ms) images, such that the measured fluorescence signals from dye-laden droplets appeared as a streak in the emulsion channel, removal and addition of dye could be directly observed. In these experiments, the measured signal from FITC dye, initially present in the dispersed phase, was diminished at the site of the pico-washer (Fig. 4b). In the emulsion channel, a decrease in the measured fluorescence of FITC corresponded with an increase in the fluorescence signal of resorufin, the dye present in the wash stream (Fig. 4c). Imaging of washed droplets off-chip confirmed that the resorufin solution was added directly to the flowing droplets and not lost to the continuous phase (Fig. S1a, b). Furthermore, quantifying the mean gray values in these droplets indicated that comparable amounts of dye were removed (CV = 4.4%) and added (CV = 3.9%) across all droplets (*N* = 100). Devices demonstrated throughputs of >1.0 kHz for pico-washing, and the input pressures for the continuous and dispersed phases were kept below 35 kPa to prevent the upstream droplet generator from operating in the jetting regime and to minimize polydispersity of the droplets entering the pico-washer. Under these operating conditions, as seen in Fig. 4a, uniform droplet sizes were observed both upstream (CV = 0.8%) and downstream (CV = 0.7%) of the pico-washer (Fig. S2).

Stable pico-washer operation was typified by a fluidic bridge connecting the wash and waste streams with a W/O droplet (Fig. 4a). Formation of this fluidic bridge required destabilization of the oil–water interfaces with an AC electric field applied to the pico-washing region: pico-washing did not occur when the field was not applied (Fig. S3a, b). This field was applied via a saltwater electrode^48^ in which the output terminals from a power source were connected with alligator clips to stainless steel inlets of the waste and wash channels. As concentrated PBS solution was used in the wash and waste streams, the conductivity of these solutions enabled efficient charge transfer, and the AC electric field was applied across the pico-washer. The AC field oscillation frequency was chosen while considering the time period during which a droplet was connected to the pico-washer, which was estimated as the time required for a droplet to move one droplet length, i.e., from the first connection (Fig. 1d, *i*) to dissociation (Fig. 1d, *iii*) from the pico-washer. For typical experimental parameters (emulsion flow rates of 2 mL/h, droplets were generated as 85 μm long plugs, and a total channel height and width maintained at 60 and 50 μm, respectively), the timescale of pico-washing was ~0.5 ms. When the AC field was applied at frequencies greater than 1/0.5 ms = 2 kHz to ensure that each droplet experienced a sufficiently high field magnitude and made fluidic contact upon approaching the pico-washer, steady and reproducible fluid exchange was observed (Fig. S4). Applying an AC field was critical in these experiments, as the use of a DC field resulted in irreproducible dye removal and droplet washing (Fig. S5).

### Operational and geometrical parameters regulate pico-washer performance

We investigated the parameter space governing the operation of a pico-washer and observed that the pressure difference between the wash and waste streams regulated the performance of pico-washing. To perform this analysis, we systematically varied the input pressures of the wash and waste streams of a given device while maintaining the input pressures of the continuous and dispersed phases. The dilution factor, which was defined as the fold change in which the concentrations of moieties originally contained within the droplet were reduced during the washing process (see Materials and Methods), was used to quantify washing performance for each set of parameters. In these experiments, we observed that increasing the input pressure on the wash stream and decreasing the input pressure on the waste stream resulted in increased dilution factors (Fig. 4d). The greatest dilution factor was observed at the largest wash pressure and smallest waste pressure, an observation consistent with the pressure difference driving fluid transfer through the droplets. It is noteworthy that the wash and waste stream input pressures could only be varied within acceptable ranges (Fig. 4e): excessively decreasing the wash stream pressure resulted in oil entering the injection channels (Fig. 4e, *i*), thereby preventing fluid transfer; excessively increasing the wash stream pressure resulted in W/O droplets forming in the emulsion channel (Fig. 4e, i*i*); and excessively decreasing the waste stream pressure resulted in oil-in-water (O/W) droplets forming in the waste stream (Fig. 4e, *iii*).

**Fig. 5:**
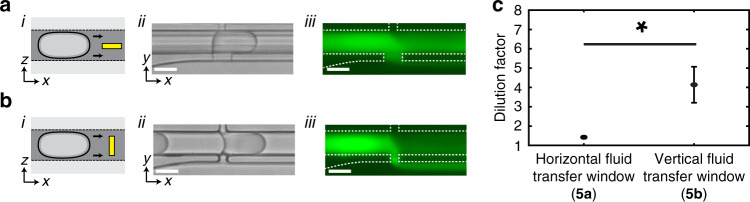
Washing efficiency depends on the geometry of the fluid transfer region. **a** (*i*) Side-profile schematic, (**a**) (*ii*) top-down transmitted light micrograph, and (**a**) (*iii*) top-down fluorescence micrograph of droplets initially laden with FITC flowing through a device with a horizontal fluid transfer region. In this device, the width of the aperture is greater than the height. **b** (*i*) Side-profile schematic, **b** (*ii*) top-down transmitted light micrograph, and (**b**) (*iii*) top-down fluorescence micrograph of droplets, initially laden with FITC, flowing through a device with a vertical fluid transfer region. In this device, the height of the aperture is greater than the width. Yellow boxes in **a** (*i*) and **b** (*i*) represent the side profiles of the fluid transfer regions in the respective devices. All scale bars represent 50 μm. **c** Washing efficiency, quantified from fluorescence images as in (**a**), (**b**) *(iii*), for each of the device geometries represented in (**a**), (**b**). Error bars represent the standard deviations determined from *n* = 4 independent devices constructed with each of the designs depicted in (**a**) and (**b**). * p < *0.05*, Mann–Whitney *U*-test.

We next investigated how the geometry of the pico-washer architecture affected washing performance by evaluating how the orientation of the fluid transfer channels influenced the measured dilution factors. To investigate this, we created pico-washers with “horizontal” fluid transfer channels (Fig. 5a, *i* and *ii*), in which the height of a channel was less than its width, as well as pico-washers with “vertical” fluid transfer channels (Fig. 5b, *i* and *ii*), for which the height of the channel was greater than the width. In both cases, at least one dimension was kept less than 15 μm to ensure a Laplace pressure high enough to immobilize the fluid interfaces in the presence of the applied pressure differential between adjacent streams. When each of these devices was operated with input pressures tuned for optimal fluid exchange, less dye removal was observed in the case of the horizontal fluid transfer region (Fig. 5a, *iii*) compared to the vertical case (Fig. 5b, *iii*). Quantitative analysis of these data revealed that the vertical pico-washer demonstrated a typical dilution factor of 4, while pico-washers with horizontal fluid transfer channels demonstrated a dilution factor of 1-1.5 (Fig. 5c).

### Quantitatively evaluating pico-washing on the subdroplet level

**Fig. 6:**
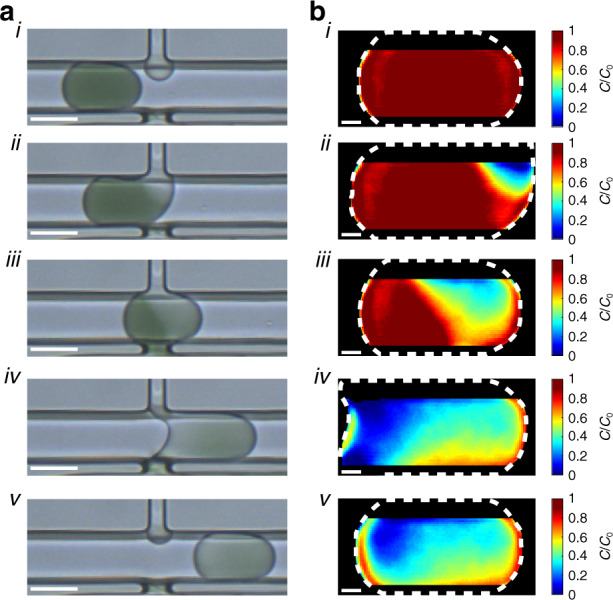
Quantitative visualization of the pico-washer in operation. **a** Transmitted light micrographs of droplets initially laden with dye. Micrographs (*i*)–(*v*) represent droplets at different stages of the washing process. Scale bars represent 50 μm. **b** Heatmaps depicting z-averaged, intradroplet dye concentrations overlaid with the outline of the respective droplet (white dashed line). Plots (*i*)–(*v*) correspond to the droplets depicted in (**a**). Scale bars represent 10 μm. Note that regions of the droplet closest to the top and bottom walls of the PDMS microchannel were not analyzed to prevent optical aberrations, due to the device fabrication process, from skewing the quantitative mapping seen in (**b**).

To aid in visualizing the pico-washing process, we developed an image processing workflow inspired by optically gated heart imaging^49^ to reconstruct videos of droplet motion with microsecond resolution. This algorithm was based on collecting many short-exposure (<10 ms) images, where each image represented a random sampling of the positions of droplets in the channel (Fig. S6). The key purpose of this workflow was to identify the position of one reference dye-laden droplet for each frame postacquisition (Fig. S6c–e) and sort the sequence of captured frames with this reference position to create an upsampled representation of a single droplet undergoing pico-washing. A representative reconstructed video depicted the operation of a pico-washer (Video S1). In this video, a droplet initially laden with dye appeared to move left-to-right toward the pico-washer and initially contacted the injection channel at the top-right portion of the droplet (Fig. 6a, *i* and *ii*). The droplet then made contact with the removal channel, and the dye was removed as the droplet progressed through the pico-washer (Fig. 6a, *iii*). Finally, the droplet broke off from the pico-washer and continued along the channel (Fig. 6a, *iv* and *v*). This reconstructed video, with 400 frames linearly distributed over the 0.5 ms pico-washing duration (Fig. S6f), depicted pico-washing with microsecond-scale resolution.

Using the reconstructed videos, we developed an additional image processing algorithm to quantitatively assess changes in dye concentration within the droplet during pico-washing (Fig. S7). In the videos, each measured intradroplet gray value was assumed to represent the z-averaged local dilution of the dye at that location. Next, we collected images of droplets with known dye dilutions in the absence of pico-washing (Fig. S7b) and created pixel-by-pixel linear models relating gray values to local dilutions of dye (Fig. S7e, f). This collection of models was then used to determine the local dye dilution, analogous to applying the Beer–Lambert law, for each pixel in each video frame. A representative video depicted this quantitative analysis of a droplet undergoing pico-washing (Video S2). Initially, the droplet was entirely red, consistent with the lack of washing (Fig. 6b, *i*). After the droplet contacted the pico-washer (Fig. 6b, *ii*), the internal regions of the droplet became progressively bluer as the droplet proceeded through the pico-washer (Fig. 6b, *iii* and *iv*). As the droplet completed pico-washing, a highly blue region was observed at the end of the droplet, indicating a spatial dependence of washing efficiency on the subdroplet level (Fig. 6b, *v*). Integrating the internal pixel values in a map corresponding to the conclusion of pico-washing in this video yielded an overall dilution factor of 3, comparable to the dilution factors obtained via long-exposure imaging (Fig. 5c).

### Operating pico-washers in series

**Fig. 7:**
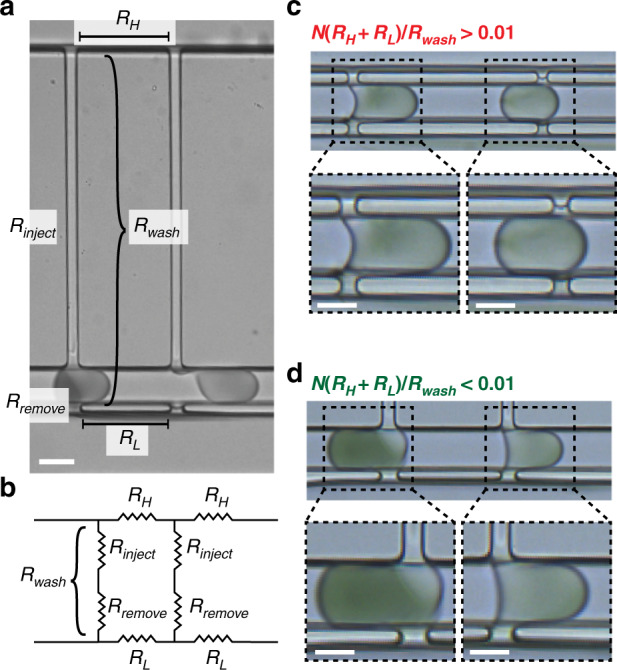
Design rule for operating multiple pico-washers in series. **a** Micrograph of a device with multiple pico-washers arranged in series and labeled with key fluidic resistances: the resistance of the washing process (*R*_wash_), with components representing the picoinjector (*R*_inject_), and the removal process (*R*_remove_); the resistance of the high-pressure wash stream between adjacent pico-washers (*R*_H_); and the resistance of the low-pressure waste stream between adjacent pico-washers (*R*_L_). **b** Circuit diagram representing the system for two pico-washers arranged in series. **c** Micrographs of droplets (initially laden with dye) in devices with multiple pico-washers in which the design rule is not satisfied. **d** Micrographs of droplets (initially laden with dye) in devices with pico-washers in which the design rule is satisfied. All scale bars represent 50 μm

We next evaluated the feasibility of operating multiple pico-washers in series, an advantage made possible by the small footprint of each pico-washer. To create such a system, we formulated and applied a design rule to inform the selection of channel dimensions and spacing of the pico-washers. In a similar microfluidic system operating many droplet generators in parallel, the fluidic resistance of the channel that connects adjacent droplet generators should be much less than the fluidic resistance within each generator to ensure uniform flow through the device^50^. We reasoned that a similar relationship was important here, and we expected that the electrical and fluidic resistances in the waste and wash streams between adjacent pico-washers should be much less than the resistance values within a given pico-washer (Fig. 7a, b) for uniform operation to occur. Mathematically, this design rule relating the internal resistance of the pico-washer (*R*_wash_), the wash stream resistance between subsequent pico-washers (*R*_H_), and the waste stream resistance between subsequent pico-washers (*R*_L_) is represented in the following equation:

$$N\frac{{\left( {R_{\mathrm{H}} + R_{\mathrm{L}}} \right)}}{{R_{{\mathrm{wash}}}}} < 0.01$$

Evaluating devices with two pico-washers, as a proof-of-concept, in series with the droplet generator revealed that this design rule was critical for proper operation. When the design rule was not satisfied, two droplets in the emulsion channel could not simultaneously connect to the two pico-washers (Fig. 7c). In this case, a fluidic connection between the second droplet and the second pico-washer only occurred once the droplet in the upstream pico-washer disconnected from that pico-washer (Video S3). To satisfy the design rule, we increased *R*_wash_ by increasing the channel length of the injection region and decreased *R*_L_ and *R*_H_ by reducing the distance between pico-washers. When these devices were operated, two droplets simultaneously connected to the two pico-washers (Fig. 7d). Reconstructed videos of these devices confirmed the simultaneous connection and operation of the two pico-washers (Video S4). Flow resistors in this successful device were chosen such that both the electrical and fluidic resistances satisfied the design rule. Quantitative analysis from long-exposure fluorescence imaging of droplets transiting the two pico-washers revealed a dilution factor of 3.1 following the first pico-washer and an overall dilution factor of 8.6 for the serial arrangement (Fig. S8).

### Incorporating solid particles during pico-washing

Finally, we performed experiments to provide preliminary insight into how the pico-washer performed when microparticles were encapsulated within the input droplets. In these experiments, nonmagnetic, 16 μm polymethylmethacrylate (PMMA) or 8 μm paramagnetic polystyrene microparticles were added to the dispersed phase, and the device was operated as previously described (Fig. S9a). As before, dilution factors >3 were realized with >1.0 kHz throughputs, and pico-washing was observed by visualizing fluid transfer of dye from the dispersed phase into the waste stream (Fig. S9e). However, in experiments with 16 μm nonmagnetic PMMA microparticles, the device could only operate on the order of minutes until one of the microparticles, which had a diameter larger than the width of the fluid transfer window within the pico-washer, became lodged between the emulsion channel and waste stream (Fig. S9b). Based on previous work that used a magnetic force to position particles within droplets^41,44,46,51^, we introduced a force on the microparticles to oppose convective flow toward the waste stream during pico-washing. Experiments were performed with 8 μm paramagnetic microparticles and a 1 in. x ½ in. x ¼ in. NdFeB permanent magnet with a surface field of 0.4 T, which was placed adjacent to the PDMS device along the side opposite of the waste stream (Fig. S9c). The microparticles were readily visible with brightfield imaging (Fig. S9d), and bead retention was calculated in subsequent image analysis by quantifying the proportion of fully formed droplets containing a microparticle in the channel region after the pico-washer relative to the proportion found in the channel prior to pico-washing, and at least 1000 droplets were analyzed in each experiment. Bead retention was 20% when the pico-washer was operated with >1.0 kHz throughput and a dilution factor of 3. When throughput was reduced to 0.5-1.0 kHz, bead retention was increased to 70% and a dilution factor of 2.5 was achieved (Fig. S9f). Integration of micrometer-scale magnets into the microchannel^52^ and optimization of device geometry could further improve these performance metrics.

## Discussion

We have demonstrated the feasibility, developed a design approach, and characterized the performance of a droplet unit operation, pico-washing, that simultaneously injects fluid into and removes fluid from droplets during flow. The fluid transfer depicted in this work results from a pressure gradient between a high-pressure wash stream and a low-pressure waste stream, with the performance of the washing process depending upon the geometry of the pico-washer. An analogous operation is well-established for just injection of fluid into moving droplets, e.g., pico-injection, and has been used to create droplet workflows for quantitative PCR^53^, pathogen testing in food^54^, and nanoparticle crystallization^55^, among other processes. However, processes for removing material from droplets or washing droplets are less established, and the technology created here is the first to demonstrate cross-flow through a moving droplet.

The first innovation that was required to realize pico-washing was development of an engineering strategy for achieving stable flow of adjacent, immiscible phases. The protocol established in this study produces PDMS devices with fluid transfer channels designed to promote three-dimensional interfacial pinning, a design choice predicated on making the wetting process, and the increase in the oil–water interface that would accompany any oil entering the waste channel, less energetically favorable. This interfacial pinning also enables spatial patterning of channel hydrophilicity by successive aqueous and oil-based surface treatments, a feature required for stable flow of each phase in the presence of the cross-droplet pressure gradient. In addition to pico-washing, the interfacial pinning and stability achieved here open a new PDMS-based fabrication avenue for other applications that require adjacent flow of immiscible phases, such as in creation of higher-order emulsions^56^ or artificial cells^57^.

Additionally, visualization of the pico-washer operation required the creation of an image processing workflow capable of quantifying the washing process with micrometer and microsecond resolution. The foundation for this workflow was the reconstruction of videos from many short-exposure images in a manner analogous to the retrospective gating applied to cardiac imaging^49^. In that application, a high frame rate depiction of a single heartbeat can be synthesized by labeling and sorting frames of a video sequence spanning multiple heartbeats. The workflow created here, in which images in a sequence are sorted by a reference position within each frame, expands this approach to droplet analyses and enables a high frame rate representation of droplet motion to be obtained with a relatively inexpensive and easily accessible camera. Quantitative mapping of local concentration changes during pico-washing is further obtained from such an image sequence by comparing the measured intradroplet gray values to those from known dilutions of dye, which is similar to a previously used approach to map spatial changes in concentration within single phase microfluidic systems^58^. When applied to droplets undergoing pico-washing, this analysis revealed that the modest washing efficiencies demonstrated by the tested architectures were limited by retention of dye at the front of the droplet. Future work aimed at improving washing efficiency should thus be directed toward incorporating process or architectural features that promote removal of dye from the front of the droplet.

Furthermore, an engineering design rule was devised for successful operation of multiple pico-washers in series, a desirable feature of this unit operation that demonstrates its potential for future integration into larger microfluidic systems. This design rule conveyed the importance of minimizing the fluidic and electrical resistances between pico-washers in the wash and waste streams relative to the resistances within a pico-washer and informed design strategies such as minimizing the distance between pico-washers, increasing the cross-sectional dimensions of the wash and waste streams, and increasing the length of the channel in the injection portion of the pico-washer. When this design rule was satisfied, several pico-washers arranged in series were observed to operate on separate droplets simultaneously (Fig. 7d). The small footprint of an individual pico-washer is one advantage of this unit operation, and future work should build upon this proof-of-concept demonstration with systems operating multiple pico-washers in series and in parallel. Given that silicon-based microfluidic systems readily achieve parallelized operation of 10^4^ droplet generators^20^, it is conceivable that future parallelized arrays of pico-washers, each operating with the kHz throughput demonstrated here, could realize MHz throughputs and match the fastest droplet generation and detection systems.

The primary aim of this work was to characterize the fabrication strategy, system parameters, and design rules for a technology capable of simultaneously injecting and removing fluid from droplets at kHz throughputs. We also performed experiments to characterize pico-washer performance when solid particles were encapsulated within the droplets. In these experiments, the pico-washer performed comparably with and without solid particles, reaching dilution factors >3 at kHz throughput, although bead retention was increased at reduced throughputs and a dilution factor of 2.5. While these results provide preliminary insight into a system not optimally designed for bead retention, the improvement that was realized suggests a possible tradeoff among washing performance, throughput, and bead retention to be optimized in future work. For example, if reduced throughput of an individual pico-washer is required for optimal bead retention, designing a parallelized array of pico-washers, as described above, offers an opportunity to increase the overall throughput while maintaining a sufficiently high residence time for each particle-containing droplet in the magnetic field. Furthermore, previous work demonstrated an avenue for increasing bead retention from 70% to >90% by increasing the magnetic content of the microparticles and the corresponding magnetic force exerted on these in the magnetic field^44^. Similar magnetic optimization, for example, by positioning the magnet closer to the emulsion channel after redesigning the channel architecture with 3D delivery vias^20^ or integrating the fabrication of nickel iron structures into the device^52^, offers one avenue for precisely tuning the magnetic force exerted on the particles during pico-washing. This optimization will be critical for achieving the >90% bead retention seen in other technologies^44,45,51^ and will be valuable for applications ranging from on-chip enzyme-linked immunosorbent assays^8,59^ to cell transfection^37,60^. Although the experiments depicted here used magnetic microparticles, strategies involving dielectrophoresis^43^, acoustophoresis^38,42^, inertial focusing^61^, or hydrogels^59^, which may facilitate fluid exchange through the solid mesh, offer alternate routes for combining pico-washing with the solid-particle-based retention of target analytes.

Taken together, this work demonstrates and characterizes a novel droplet unit operation, as well as the key fabrication, quantification, and design innovations required to realize it. The pico-washer created here likely represents an early iteration of potential technology for on-demand droplet washing and is capable of functioning as a standalone device. We thus expect this work to contribute to the continued emergence of novel droplet unit operations that will make the development of multistep biological and chemical assays a more widespread reality.

## Materials and methods

### Microfluidic device fabrication

Microfluidic devices were fabricated at The Singh Center at The University of Pennsylvania. Photomasks were designed using AutoCAD 2018 (Autodesk, Inc.) and created by writing the design files on chrome-coated soda lime photomasks (AZ1500) using a DWL 66+ mask writer (Heidelberg Instruments) with a 10 mm write head. Following exposure, the photomasks were developed in AZ 300 MIF (EMD Performance Materials Corp.) for 1.5 min, etched in Chromium Etchant 1020AC (Transene) for 2.5 min, and residual photoresist was removed via sonication for 10 min in Microposit Remover 1165 (Dow) at 60 °C. A SUSS MicroTec MA6 Gen3 mask aligner was used to lithographically define features of SU8 (Kayaku Advanced Materials) on silicon wafers (University Wafer) to produce molds for soft lithography.

The two PDMS halves of a given device were produced by curing a 15:1 (base:crosslinker) mixture of PDMS (Sylgard 148) in different SU8 molds at 95 °C on a level hotplate. To bond the two PDMS halves together, the PDMS surfaces were first activated via oxygen plasma treatment in a barrel asher (Anatech SCE-106). Following activation, the two PDMS pieces were immediately aligned with a mask aligner (ABM 3000HR), brought into contact, and allowed to bake on a hotplate at 100 °C for at least 10 min. The resulting device consisted of robustly bonded PDMS that did not exhibit leakage during device operation.

Following device construction, the waste and emulsion channels were rendered hydrophilic and hydrophobic, respectively, by successively flowing different solutions through each. First, 1% poly(vinyl alcohol) (PVA; Sigma, average molecular weight 85,000–124,000, 87–89% hydrolyzed) in Millipore water was flowed into the waste channel for 10 min. During this time, air was injected into the emulsion channel at 60 mL/h to prevent the PVA solution from contaminating other channels. Following this surface treatment, air was injected into the waste channel to remove the PVA solution. Next, 0.05% trichloro(1H,1H,2H,2H-perfluorooctyl)silane (Sigma) in HFE-7500 (Oakwood Chemical) was flowed through the emulsion channel to render it hydrophobic. As before, air was injected at a rate of 60 mL/h through the waste channel to prevent silane contamination. Following 20 min of silanization, the entire device was thoroughly washed with HFE-7500 and immediately used in an experiment.

### Device operation and imaging

A custom-built pressure-driven flow apparatus connected to compressed nitrogen was used to deliver the four fluidic input streams to the prepared devices. Two of these streams represented the dispersed and continuous phases of the emulsion: aqueous droplets were formed in a continuous phase of fluorinated oil (Bio–Rad, QX200™ Droplet Generation Oil for EvaGreen). Input pressures for the dispersed and continuous phases were adjusted between 14 and 35 kPa, which produced an emulsion flow rate of 2 mL/h and a droplet volume fraction of ~0.5. The input pressure of the aqueous wash stream was adjusted between 7 and 35 kPa, and the output of this channel was closed to ensure that the wash fluid was distributed entirely to the pico-washer(s) in the microfluidic device. The input to the waste stream was adjusted between 7 and 21 kPa. The dispersed phase, the wash stream, and the waste stream consisted of 2x PBS (Research Products International). All input pressures were adjusted as necessary to achieve consistent operation between experiments, and all flow rates were determined by collecting fluid from the desired output over a set amount of time.

The saltwater electrode was charged using an AFG3102C function generator (Tektronix) outputting a 5 V_pp_, 100 kHz sinusoidal signal that was amplified 20-fold by an A-301 HS high-voltage amplifier (A.A. Lab Systems, Ltd.). The output from the high-voltage amplifier was connected via alligator clips to the metal tubing at the input of the wash and waste streams and verified to be 100 V_pp_ at 100 kHz with a voltage probe. Experiments were run on the stage of a Leica DM4B Upright Research Microscope. All imaging was performed with a FLIR Grasshopper3 CMOS camera (GS3-U3-23S6C-C) and a 10x/0.30NA objective. Transmitted light images were acquired with <10 μs exposures. Long-exposure fluorescence images, used to quantify washing efficiency averaged over many droplets, were acquired with ~400 ms exposures.

### Visualization and quantification of liquid exchange via long-exposure imaging

The degree of fluid turnover during the droplet washing process was visualized by adding two spectrally distinct fluorescence dyes to the dispersed phase and wash stream and performing long-exposure fluorescence imaging. For these experiments, a solution of fluorescein isothiocyanate–dextran (FITC; Sigma, average molecular weight 10,000), diluted in 2x PBS to a final concentration of 25 μg/mL, was used as the dispersed phase. Resorufin, produced by diluting QuantaRed™ Enhanced Chemifluorescent HRP Substrate (Thermo Scientific) and Pierce™ High Sensitivity Streptavidin-HRP (Thermo Scientific) in 2x PBS, was used as the wash stream.

Washing efficiency was calculated from three long-exposure images of FITC, the dye initially present in the dispersed phase: an image of the emulsion channel during pico-washing (Fig. S3a); an image of the emulsion channel without any washing (Fig. S3b); and a background image of the PDMS device away from the fluidic channels (Fig. S3c). These three images were acquired for each device. All images were aligned, and the pixels corresponding to the emulsion channel were integrated over the cross-section of the channel in each image to produce three intensity profiles spanning the length of the emulsion channel (Fig. S3d): *I*_wash_, corresponding to the device in operation; *I*_cntl_, corresponding to the device running without an applied electric field; and *I*_back_, corresponding to the background fluorescence signal. A normalized intensity profile, *I(x)*, was then calculated according to the following formula, where *x* refers to the dimension that runs along the length of the emulsion channel (Fig. S3e).

$$I\left( x \right) = \frac{{I_{{\mathrm{wash}}}\left( x \right) - I_{{\mathrm{back}}}(x)}}{{I_{{\mathrm{cntl}}}\left( x \right) - I_{{\mathrm{back}}}(x)}}$$

The normalized intensity measured at a given pixel was taken to be proportional to the fraction of time that a droplet was present at the corresponding position in the channel (*p*_droplet_) and the concentration of dye that was uniformly distributed within each droplet (*C*_dye_):

$$I\sim p_{{\mathrm{droplet}}}C_{{\mathrm{dye}}}$$

The proportion of time the droplet was present at a given position was estimated from the droplet plug length (*L*_drop_) and spacing between droplets (*L*_space_) (Fig. S3f).

$$p_{{\mathrm{droplet}}}\sim \frac{{L_{{\mathrm{drop}}}}}{{L_{{\mathrm{drop}}} + L_{{\mathrm{space}}}}}$$

To quantitatively describe washing from long-exposure images of dye-laden droplets, the dilution factor (DF) was calculated from the following formula, where *C*_pre_ and *C*_post_ are the concentrations of some species in a droplet before and after washing, respectively.

$${\mathrm{DF}} = \frac{{C_{{\mathrm{pre}}}}}{{C_{{\mathrm{post}}}}}$$

By averaging *I(x)* over many pixels before (*I*_pre_) and after (*I*_post_) the washer and substituting into the previous equation, the dilution factor was calculated from quantities measured experimentally, as follows.

$${\mathrm{DF}} = \frac{{\left\langle {I_{{\mathrm{pre}}}} \right\rangle \frac{{L_{{\mathrm{drop}},{\mathrm{post}}}}}{{L_{{\mathrm{drop}},{\mathrm{post}}} + L_{{\mathrm{space}},{\mathrm{post}}}}}}}{{\left\langle {I_{{\mathrm{post}}}} \right\rangle \frac{{L_{{\mathrm{drop}},{\mathrm{pre}}}}}{{L_{{\mathrm{drop}},{\mathrm{pre}}} + L_{{\mathrm{space}},{\mathrm{pre}}}}}}}$$

When calculating the dilution factor for two pico-washers arranged in series, the dilution factor after the first pico-washer was calculated by first averaging *I(x)* over all pixels between the two pico-washers. While some change in droplet volume was observed during operation, the input fluidic pressures were tuned to minimize this change during the pico-washing process.

### Droplet video reconstruction and visualization of liquid exchange via short-exposure imaging

Pico-washing was visualized with short exposure, transmitted light imaging of droplets generated with concentrated food coloring (Clover Valley) added to the dispersed phase. These images were used to construct videos of the washing process according to a custom image processing algorithm (Fig. S6). First, hundreds to thousands of images of the device in operation were acquired (Fig. S6a). Next, for each image, the position of a reference droplet was identified by either *(i*) image segmentation via intensity-based thresholding and then determining the centroid of the object (Fig. S6b) or (*ii*) by finding a local minimum in the sum-square difference in gray values between a reference image of a droplet and a subimage defined at each pixel position along the length of the channel (Fig. S6c, d). In the latter, a subimage (Fig. S6c, *ii*) defined for the *x*-th pixel in the channel was a cropped image of the channel region (Fig. S6c, *i*) with dimensions that matched the reference image (Fig. S6c, *iii*) and with the first column of gray values matching those of the *x*-th column in the channel image. That is, the gray value of the *i*-th row and *j*-th column of the subimage, *g*, corresponded to the gray value of the *i*-th row and *(j* *–* *1* *+* *x)*-th column of the channel image, *c*.

$$g_{i,j}(x)\sim c_{i,j - 1 + x}$$

The value of the sum-square difference, *E*(*x*), was calculated for each pixel position by summing the squared differences between all *N* pairs of gray values in the subimage and reference image, *G*_ref_.

$$E(x)\sim \mathop {\sum }\limits_k^N (g_k(x) - G_{{\mathrm{ref}},k})^2$$

In this case, the position of a droplet was determined by finding the local minimum of *E*(*x*) (Fig. S6e). Once the position of a reference droplet within some bounds was identified for each frame, the sequence of images was reordered by these positions to produce a representation of droplet movement along the channel.

To convert the transmitted light videos of pico-washing into quantitative heatmaps of intradroplet dye dilution, the gray values in the droplet from each frame were compared to the gray values contained within droplets of known dilutions of the dye (Fig. S7). First, droplets were formed without the presence of an electric field and encapsulated with known dilutions of the food coloring dye solution used previously (Fig. S7a). Images were converted to grayscale by averaging the R-G-B values for each pixel, the images were segmented, and gray values for each intradroplet pixel location were stored. These values were averaged on a pixel-by-pixel basis over 500 image frames for each known dye dilution to achieve a reference image depicting the average gray value at each pixel location for that dye dilution (Fig. S7b, c). These reference images enabled construction of a linear model for each pixel location to convert a measured gray value to a local z-averaged dye dilution, which is comparable to applying the Beer–Lambert Law (Fig. S7d, e). All image processing and data analysis were performed using custom scripts written in ImageJ and MATLAB 2021a (MathWorks, Inc.).

### Incorporating beads into droplets

To evaluate device operation with solid particles encapsulated within droplets, 16 μm nonmagnetic polymer microspheres (Automate Scientific Inc) and 8 μm paramagnetic iron oxide-coated polystyrene microspheres (Spherotech, FCM-8052-2) were incorporated into droplets. In these experiments, the density of the aqueous solution was matched to the density of the microparticles by mixing 2× PBS with OptiPrep Density Gradient Medium (Sigma–Aldrich). In the experiments with magnetic beads, a 1 in. x ½ in. x ¼ in. N52 NdFeB permanent magnet (K&J Magnetics, Inc. # BX084-N52) was placed adjacent to the microfluidic device and on the side opposite the waste channel (Fig. S9c). Retention of beads in droplets was quantified by dividing the proportion of droplets containing a microparticle after pico-washing by the proportion of fully formed droplets containing a microparticle upstream of the pico-washer. At least 1000 droplets were analyzed in each experiment.

## Supplementary information

Video S1

Video S2

Video S3

Video S4

Supplemental Information
