# Supplementary material for: Pico-washing: simultaneous liquid addition and removal for continuous-flow washing of microdroplets
Source: Microsyst Nanoeng. 2022 Apr 29;8:46. doi: 10.1038/s41378-022-00381-3 (PMC9050730; doi:10.1038/s41378-022-00381-3)
Supplement: Supplementary file 5 — Supplemental Information [file 41378_2022_381_MOESM5_ESM.docx]

**Supplementary Information for:**

**Pico-washing: liquid exchange for continuous-flow washing of microdroplets**

**Michael J. Siedlik^1^ and David Issadore^,1,2*^**

Departments of ^1^Bioengineering, ^2^ Electrical and Systems Engineering

University of Pennsylvania, Philadelphia, PA 19104

**Sample data and image processing code**

Example data and programming files for the computational workflow described here are freely available at the Harvard Dataverse (<https://dataverse.harvard.edu/>) in the Pico-washing dataset (<https://doi.org/10.7910/DVN/GBBDSG>), or upon requrest from the authors. Example data is provided with the following MatLab scripts.

1. *A_ReconstructVideo.m*

Script for creating the reconstructed videos of droplet motion, as in **Figures S5** and **Video S1**.

1. *B_CreateControlDilutionImages.m*

Script for creating reference images of the average intra-droplet grey values for known dilutions of dye, as used in **Figure S6c** and **Video S2**.

1. *C_QuantitativeMapping.m*

Script for creating quantitative maps of local changes in dye concentration, as in **Figure S6** and **Video S2**.

**Supplementary Figures and Supplementary Figure Legends**


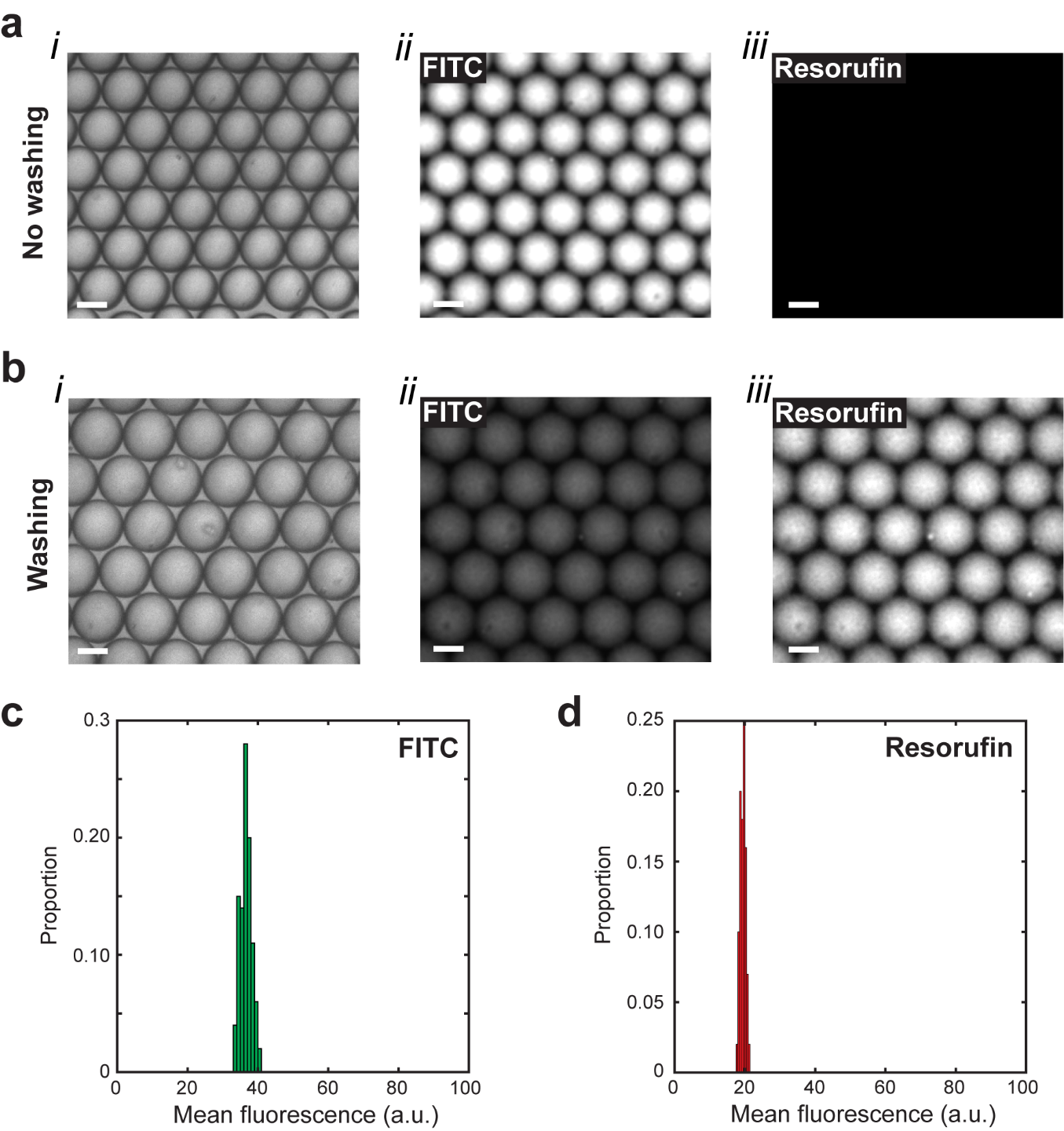


**Figure S1: Dye is removed from and added to droplets during pico-washer operation.** (a, *i*) Transmitted light micrograph of collected droplets, (a, *ii*) fluorescence micrograph of dye initially contained within the droplets, and (a, *iii*) fluorescence micrograph of dye initially contained in the wash stream when the saltwater electrode is not activated and pico-washing does not occur. (b, *i*) Transmitted light micrograph of collected droplets, (b, *ii*) fluorescence micrograph of dye initially contained with the droplets, and (b, *iii*) fluorescence micrograph of dye initially contained in the wash stream following pico-washing. (c) Histogram of mean gray values (CV = 4.4%; N = 100 droplets) contained within the droplets imaged in (b, *ii*). (d) Histogram of mean gray values (CV = 3.9%; N = 100 droplets) contained within the droplets represented in (b, *iii*). All scale bars represent 50 μm.


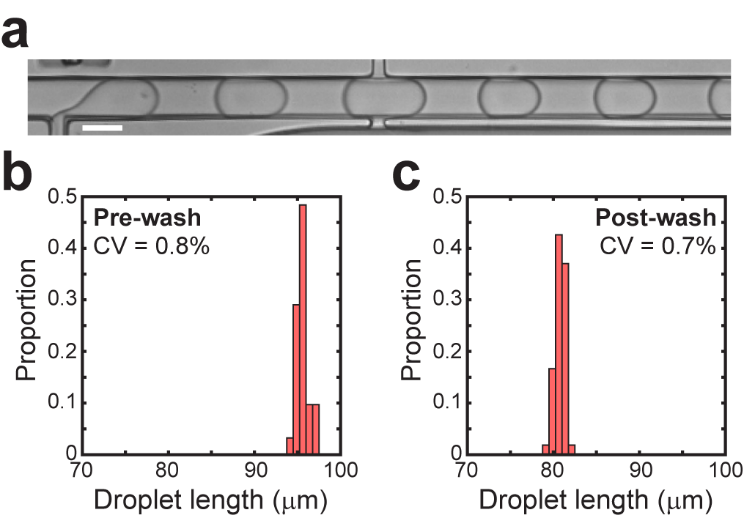


**Figure S2: Droplet sizes are highly uniform prior to and following pico-washing.** (a) Transmitted light image of droplets traversing a pico-washer in the device represented in **Fig. 4**. (b) Histogram of plug lengths of fully formed droplets upstream of the pico-washer (CV = 0.8%; N = 31 droplets). (c) Histogram of droplet plug lengths immediately downstream of the pico-washer (CV = 0.7%; N = 54 droplets). Scale bar represents 50 μm.


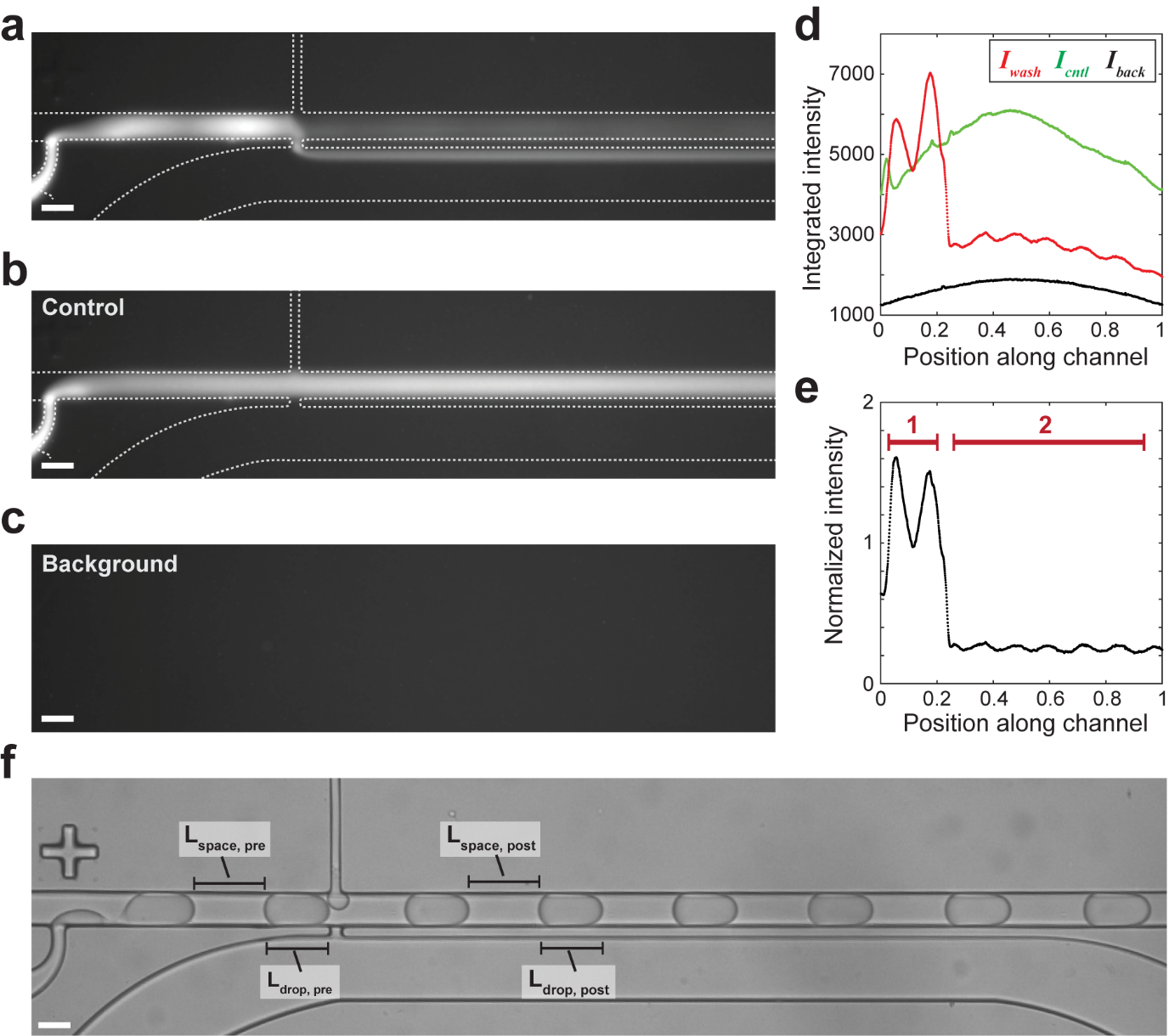


**Figure S3: Washing efficiency is calculated from a long-exposure fluorescence image of the device in operation, a control image of the droplets without the application of the saltwater electrode, and a background image.** (a) Fluorescence micrograph of dye initially contained within the droplets during pico-washer operation. The white, dashed line provides a reference outline of the channels. This image is comparable to Figure 4b. (b) Fluorescence micrograph of device in operation without the application of the saltwater electrode. In this case, there is no fluid transfer. (c) Fluorescence micrograph of the background signal arising from the optical setup and PDMS device. (d) Plot depicting the integrated channel intensity profiles, *I_wash_*, *I_cntl_*, and *I_back_*, corresponding to the images depicted in (a), (b), and (c), respectively. (e) Normalized intensity profile along the length of the channel. Regions 1 and 2 correspond to the normalized intensities before and after the pico-washer, respectively. (f) Transmitted light image highlighting the lengths used to correct for small changes in droplet length in the calculation of the dilution factor. All scale bars represent 50 μm.


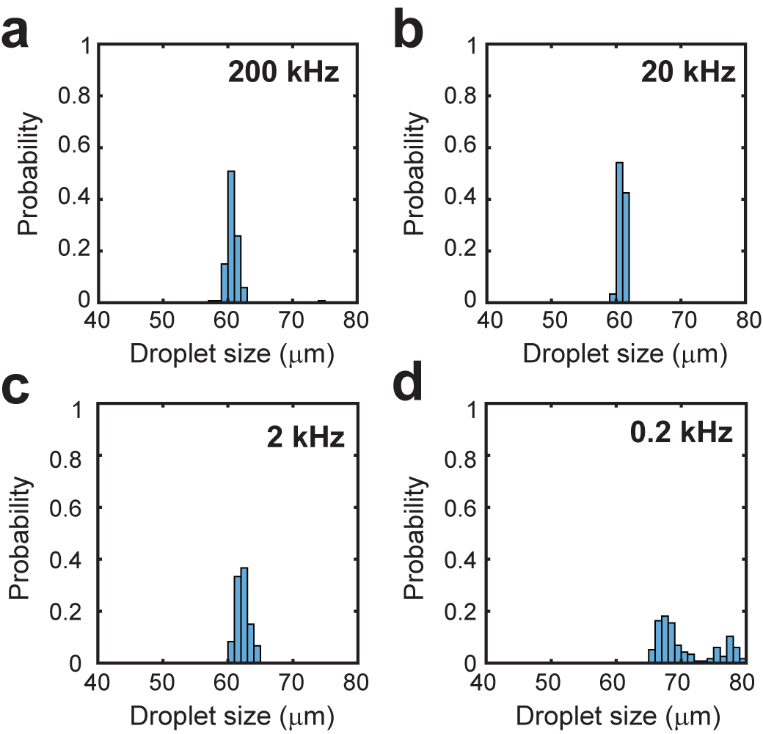


**Figure S4: Pico-washing produces a narrow distribution of droplet sizes for sufficiently high AC field frequencies.** Histograms depicting distributions of droplet sizes in an example experiment after pico-washing for applied AC field frequencies of (a) 200 kHz, (b) 20 kHz, (c) 2 kHz, and (d) 0.2 kHz.


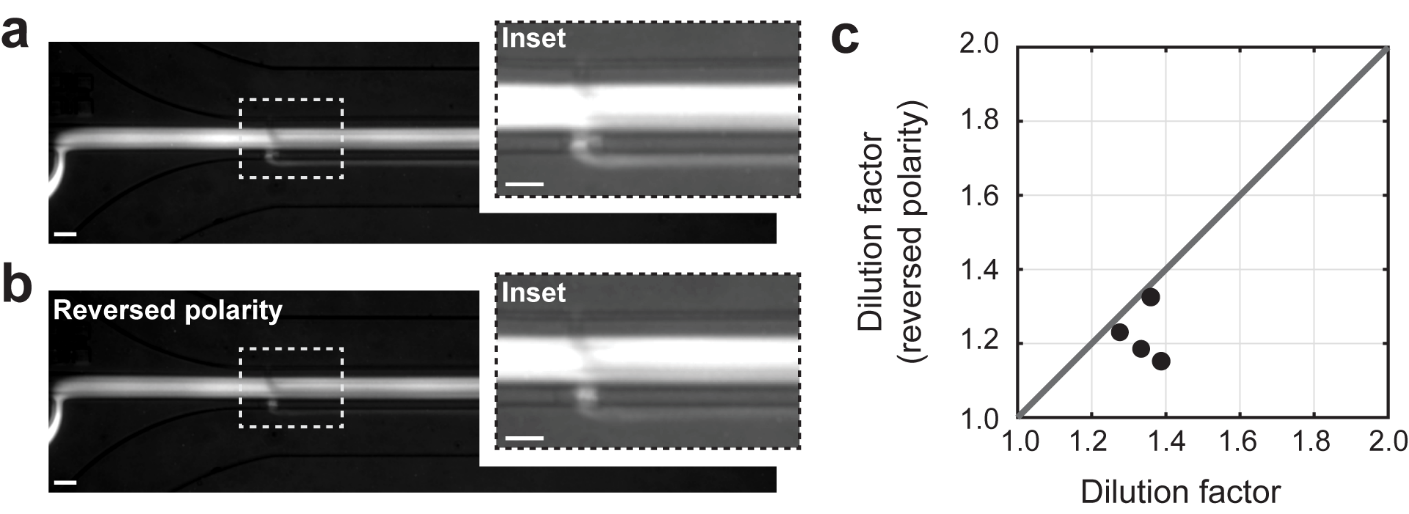


**Figure S5: An AC field provided by the saltwater electrode was necessary for reproducible device operation.** (a) Fluorescence micrograph of dye initially contained within the droplets during pico-washer operation. (b) Fluorescence micrograph of dye initially contained within the droplets during pico-washer operation, with the polarity of the field reversed relative to (a). Note the diminished dye signal in the waste stream relative to (a). (c) Plot of the calculated dilution factor for individual devices without and with reversing the polarity of the DC field. Each data point represents one device in operation: the x value represents the dilution factor calculated when the device was set up in a configuration with the positive terminal connected to the wash stream; the y value represents the dilution factor calculated in the same device after the polarity of the DC field was reversed. All scale bars represent 50 μm.


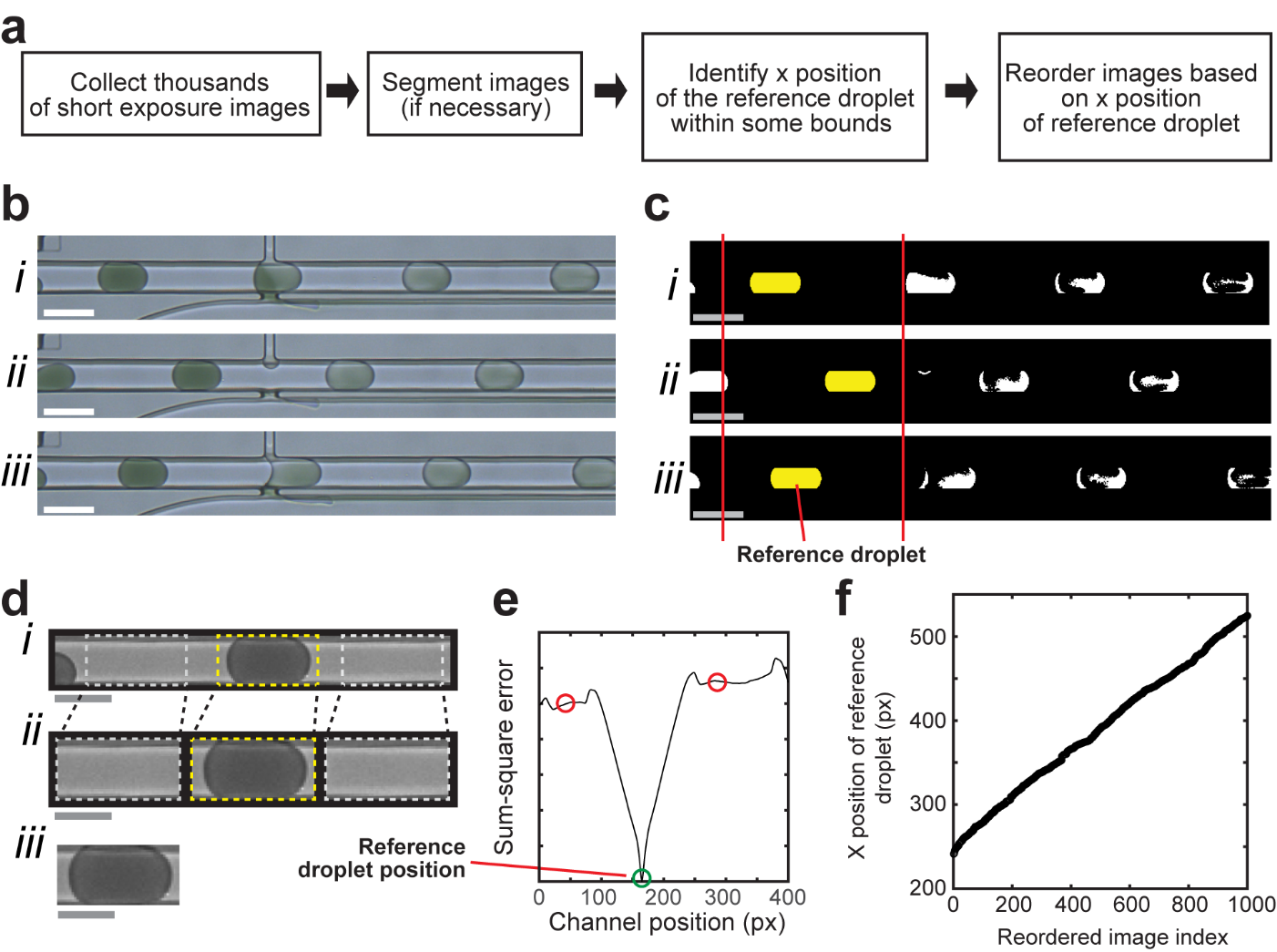


**Figure S6: Image processing workflow to visualize droplet motion with microsecond resolution.** (a) Process schematic of image processing workflow. (b) Three representative transmitted light images of the device in operation. (c) Segmented images highlighting the reference droplet (yellow) obtained by intensity-based thresholding of the corresponding images in (b). (d) Representative transmitted light images used to determine the reference droplet position by comparing an image of a known droplet. (*i*) Transmitted light image of the channel region immediately upstream of a pico-washer. (*ii*) Three example cropped regions of (*i*), each corresponding to a different sub-image of the channel. (*iii*) A reference image of a droplet. (e) Plot depicting the sum-square difference between pixel values in the reference image (d, *iii*) and each sub-image formed by cropping the channel at progressively downstream channel positions. The circles highlight values for the three sub-images represented in (d, *ii*). (f) Plot of the x positions of the reference droplets in the reordered image sequence of a 1,000-frame video. All scale bars represent 50 μm.


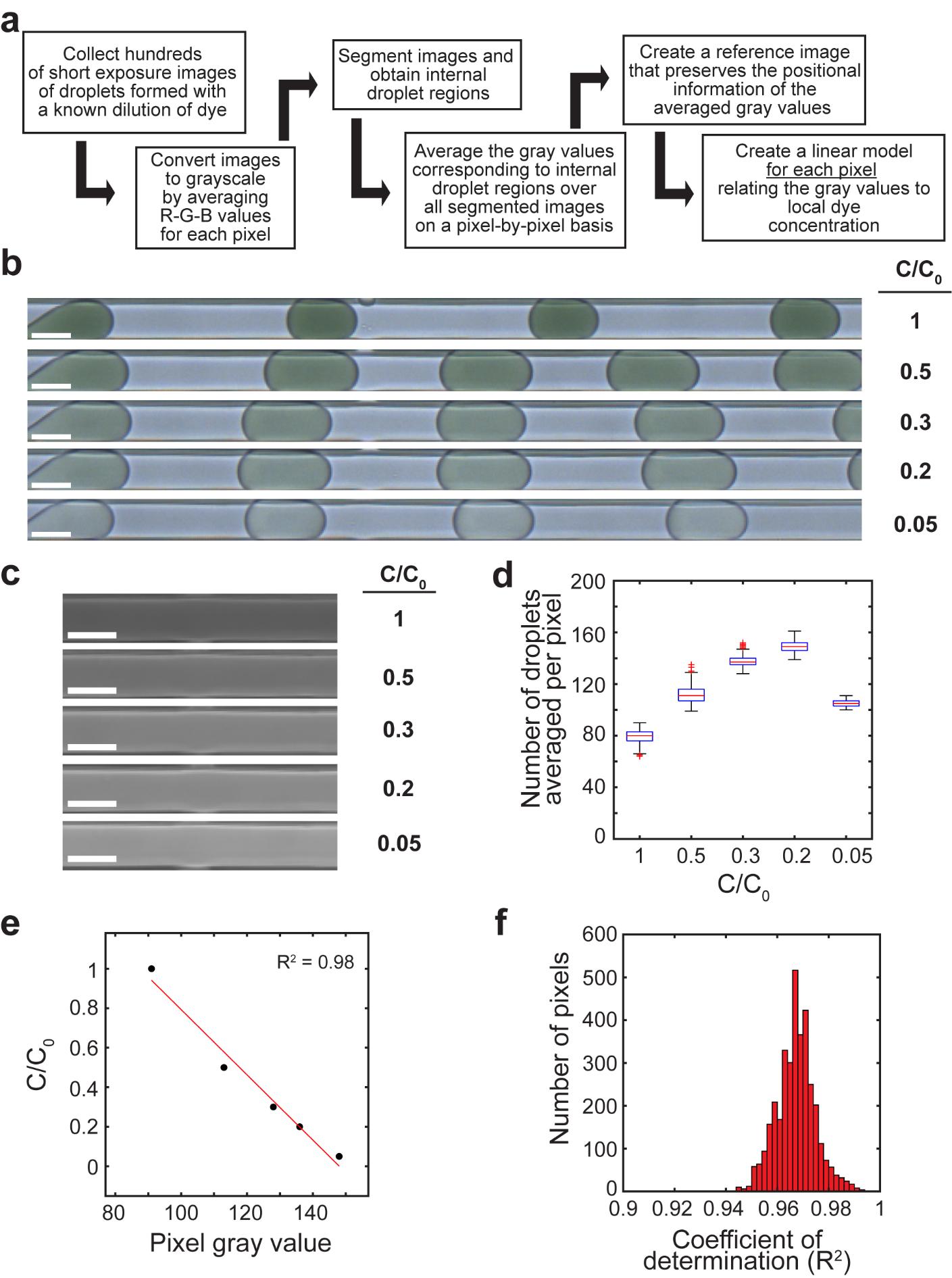


**Figure S7: Image processing workflow to quantify local z-averaged dye concentrations with microsecond and micrometer resolution during pico-washing.** (a) Process schematic of image processing workflow. (b) Short exposure images representing the formation of droplets with known dilutions of dye and the flow of these droplets through the device without pico-washing. (c) Reference images obtained by averaging intra-droplet gray values present at each pixel location. (d) Boxplots representing the distribution of droplets averaged by pixel for each known dilution. (e) Representative overlay of data points (black) with a linear model (red) of gray values vs. known dye concentrations for one pixel location. (f) Distribution of coefficients of determination for all pixel locations. All scale bars represent 50 μm.


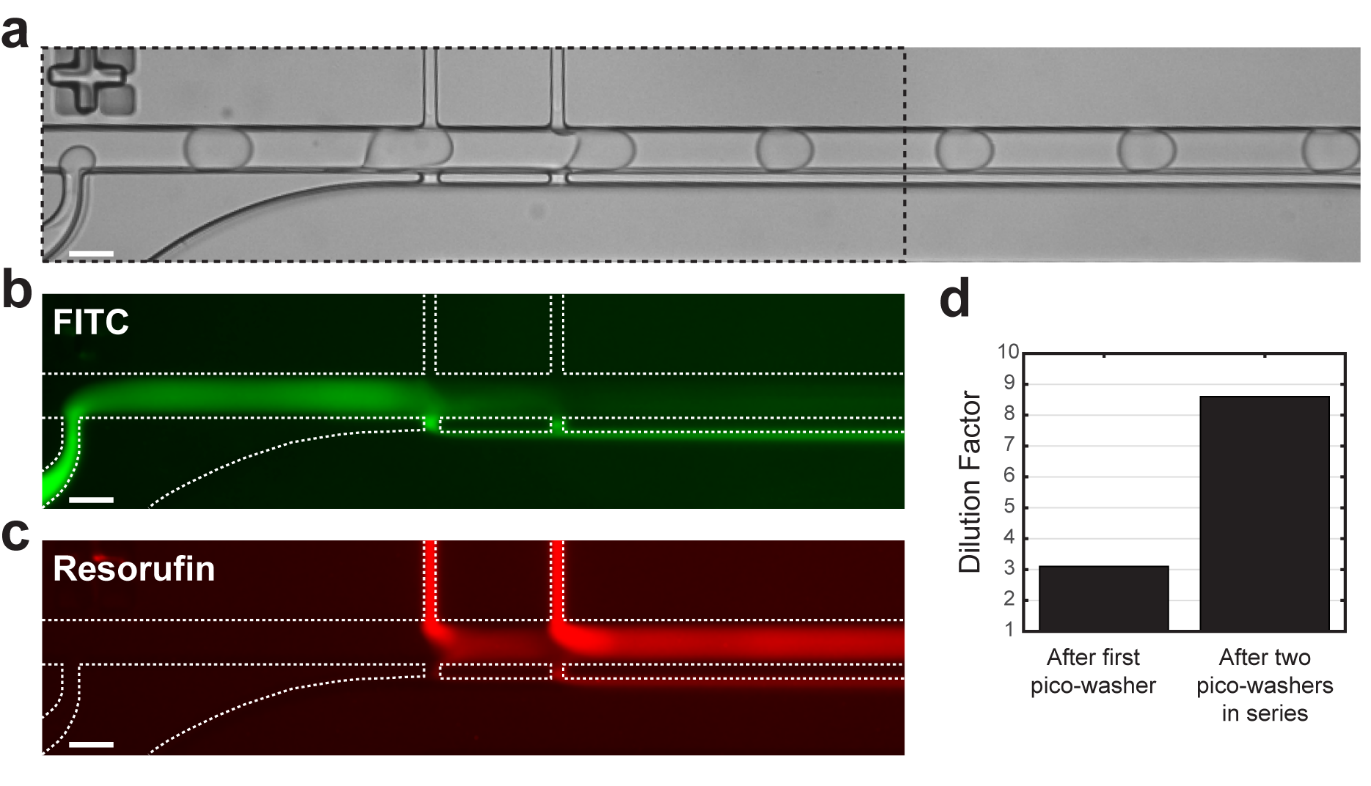


**Figure S8: Arranging two pico-washers in series compounds the washing performance of each pico-washer.** (a) Transmitted light micrograph of two pico-washers arranged in series. (b) Fluorescence micrograph of dye initially present within the droplets. (c) Fluorescence micrograph of dye added to droplets during the washing process. (a)-(c) all correspond to the same device. The black dashed box in (a) represents the region depicted in (b) and (c). (d) Bar plot representing the dilution factor calculated after the first pico-washer and the dilution factor calculated after the two pico-washers arranged in series. All scale bars represent 50 μm.


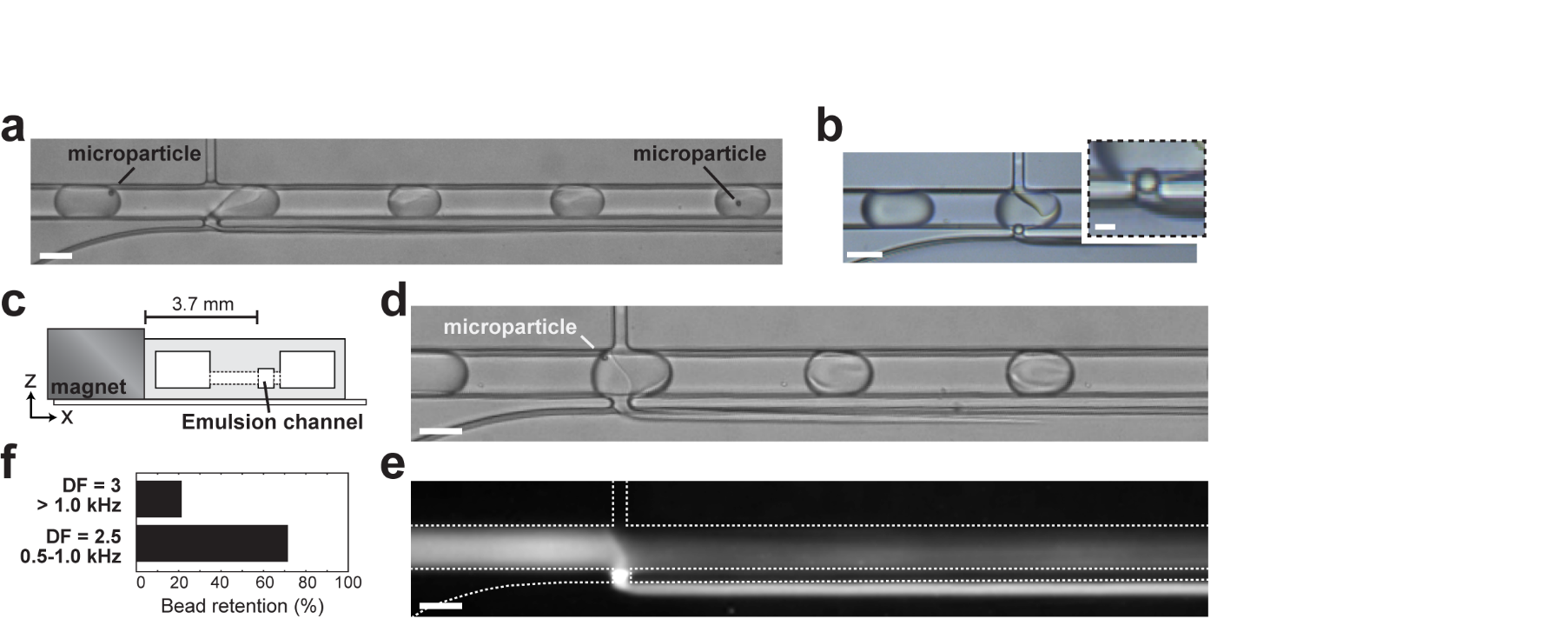


**Figure S9: Pico-washer performance with droplets containing microparticles.** (a) Transmitted light micrograph of droplets containing 8 μm polystyrene microparticles transiting a pico-washer. (b) Transmitted light micrograph of droplets containing 16 μm non-magnetic polymethylmethacrylate microparticles transiting a pico-washer. Inset depicts a microsphere lodged in the fluid transfer aperture between the emulsion channel and waste stream. (c) Schematic of the experimental setup used to test the retention of 8 μm paramagnetic polystyrene microparticles in droplets during pico-washing. (d) Transmitted light micrograph of two pico-washers arranged in series. (e) Fluorescence micrograph of dye initially present within the droplets. (f) Bar plot depicting the retention of 8 μm paramagnetic polystyrene microparticles in droplets for two washing performances. (d) and (e) correspond to the device represented by the bottom bar in (f). All scale bars represent 50 μm, except for the scale bar representing 10 μm in the inset of (b).

**Supplementary Videos and Supplementary Video Legends**

**Video S1**: Reconstructed video depicting droplet transit through a pico-washer.

**Video S2**: Quantitative mapping of the pico-washing process. Colors represent the change in local z-averaged dilutions of dye. This quantitative mapping is based on the reconstructed video depicted in Video S1.

**Video S3**: Reconstructed video depicting droplet transit through two pico-washers arranged in series when the design rule was not satisfied.

**Video S4**: Reconstructed video depicting droplet transit through two pico-washers arranged in series when the design rule was satisfied.
